# Characterization of Hydroxyapatite Film Obtained by Er:YAG Pulsed Laser Deposition on Sandblasted Titanium: An In Vitro Study

**DOI:** 10.3390/ma15062306

**Published:** 2022-03-20

**Authors:** Lin Ma, Min Li, Satoshi Komasa, Sifan Yan, Yuanyuan Yang, Mariko Nishizaki, Liji Chen, Yuhao Zeng, Xin Wang, Ei Yamamoto, Shigeki Hontsu, Yoshiya Hashimoto, Joji Okazaki

**Affiliations:** 1 Department of Removable Prosthodontics and Occlusion, Osaka Dental University, 8-1, Kuzuha hanazono-cho, Hirakata-shi, Osaka 573-1121, Japan; ma-l@cc.osaka-dent.ac.jp (L.M.); komasa-s@cc.osaka-dent.ac.jp (S.K.); yan-z@cc.osaka-dent.ac.jp (S.Y.); yang-y@cc.osaka-dent.ac.jp (Y.Y.); nishizaki-m@cc.osaka-dent.ac.jp (M.N.); zeng-y@cc.osaka-dent.ac.jp (Y.Z.); wang-xi@cc.osaka-dent.ac.jp (X.W.); joji@cc.osaka-dent.ac.jp (J.O.); 2 Department of Orthodontics, Osaka Dental University, 8-1, Kuzuha hanazono-cho, Hirakata-shi, Osaka 573-1121, Japan; chen-li@cc.osaka-dent.ac.jp; 3 Department of Biomedical Engineering, Faculty of Biology-Oriented Science and Technology, Kindai University, 930 Nishimitani, Kinokawa 649-6493, Japan; ei@waka.kindai.ac.jp (E.Y.); hontsu@waka.kindai.ac.jp (S.H.); 4 Department of Biomaterials, Osaka Dental University, 8-1, Kuzuha Hanazono-cho, Hirakata 573-1121, Japan; yoshiya@cc.osaka-dent.ac.jp

**Keywords:** Er:YAG laser, pulsed laser deposition, hydroxyapatite coating, titanium implant

## Abstract

The surface of titanium (Ti) dental implants must be modified to improve their applicability, owing to the biological inertness of Ti. This study aims to use sandblasting as a pretreatment method and prepare a hydroxyapatite (HA) coating on Ti to improve its biocompatibility and induce bone bonding and osteogenesis. In this paper, sandblasted Ti discs were coated with α-tricalcium phosphate (α-TCP) via Er:YAG pulsed laser deposition (Er:YAG-PLD). An HA coating was then obtained via the hydrothermal treatment of the discs at 90 °C for 10 h. The surface characteristics of the samples were evaluated by SEM, SPM, XPS, XRD, FTIR, and tensile tests. Rat bone marrow mesenchymal stem cells were seeded on the HA-coated discs to determine cellular responses in vitro. The surface characterization results indicated the successful transformation of the HA coating with a nanorod-like morphology, and its surface roughness increased. In vitro experiments revealed increased cell attachment on the HA-coated discs, as did the cell morphology of fluorescence staining and SEM analysis; in contrast, there was no increase in cell proliferation. This study confirms that Er:YAG-PLD could be used as an implant surface-modification technique to prepare HA coatings with a nanorod-like morphology on Ti discs.

## 1. Introduction

Titanium (Ti) and its alloys have developed as commonly utilized materials in the field of oral implantation because of their excellent chemical stability, mechanical properties, and biocompatibility [1,2]. Nevertheless, because Ti and its alloys form a mechanical lock with tissues, the surface of these materials presents a certain degree of biological inertness. Studies indicate that surface modification may provide Ti surfaces with biological functionality, and research into the formation of early osseointegration with bone tissue is well advanced [3]. Common methods of Ti surface modification include physical, chemical, and biochemical methods, and the success of these procedures can be observed as alterations in surface morphology, chemical composition, surface roughness (Ra), and hydrophilic characteristics [4]. Implant modification can promote the surface adhesion, proliferation, differentiation, and mineralization of bone cells and shorten the implantation cycle of the implants, thereby achieving earlier and more stable osseointegration between the device and bone tissues [5]. Sandblasting is an excellent surface pretreatment method in engineering; it uses compressed air to form high-speed jets and spray particles of different sizes on the surface of an implant, in order to change its Ra and surface area, ultimately promoting cell adhesion, cell proliferation, and osseointegration capacity [6,7]. The resulting material shows extensive application prospects in the field of orthopedic biology.

Several researchers have identified hydroxyapatite (HA) as a bioactive substance with good biocompatibility and osteoconductivity [8]. HA has a similar composition to bone tissue, has corrosion resistance, has excellent osteogenic and osseointegration properties, and is also biodegradable [9]. Nano-HA shows stable performance under a physiological environment and high chemical activity, which is beneficial in increasing its biological activity; moreover, the large surface area of the material is conducive to cell attachment and growth [10]. Nano-HA secretes a variety of osteogenic differentiation factors and provides crystal nuclei for bone conduction during bone cell calcification and bone formation. The synthesis methods of nano-HA mainly include water-phase synthesis, chemical precipitation, hydrothermal reaction, the sol–gel method, natural combustion, and microemulsion, among others [11]. However, HA has poor mechanical properties; thus, it is not a load-bearing implant material. Ti and other alloys show excellent mechanical properties, but they are biologically inert. Thus, the preparation of HA-based bioceramic coatings on the surface of Ti and its alloys has important theoretical and practical significance [12].

The erbium-doped yttrium aluminum garnet (Er:YAG) laser is a hydrodynamic biological laser system [13,14] that is widely used in clinical settings for oral enamel repair, skin freckle removal, etc. [15,16]. When a target is irradiated by the laser, the laser energy is transferred to the coaxial water–air mixture. The activated water mist produces water molecules with ultrahigh energy. These water molecules act on the target so that the particles are separated into smaller particles and deposited on a Ti plate [17,18]. A previous study [19] revealed that Er:YAG pulsed laser deposition (PLD) can be used to coat α-tricalcium phosphate (α-TCP), β-TCP, HA, and other materials on Ti plates; specifically, α-TCP could be successfully attached to these plates. However, the literature indicates that α-TCP lacks osteoinductive ability [20]. Researchers often employ the hydrothermal method to convert α-TCP to HA [21,22,23,24,25,26]. In addition, the hydration rate of α-TCP is affected by various factors, such as solvent type, reaction temperature and binding, and initial pH. The reaction processing conditions also largely determine the microstructure of HA. [27,28]. In a previous study, for example, HA was prepared at temperatures of 60, 70, 80, and 90 °C, and the complete conversion of α-TCP to HA was observed at 90 °C. [29]

After the implantation, attachment, proliferation, differentiation, and mineralization of cells on the implant surface, all of which directly affect implant osseointegration [22,30], the cellular responses of the material must be evaluated. Previous studies used these cells to evaluate the cellular activity and osteogenic ability of the material [23,31,32,33].

This study aimed to use sandblasting as a pretreatment method and prepare HA coatings on Ti discs via Er:YAG-PLD. The surface characteristics and cell viability of the samples were then evaluated. The results of this work may provide a novel approach to improve the biocompatibility of bone implants and induce their bone bonding and osteogenesis.

## 2. Materials and Methods

### 2.1. Fabrication of the Hydroxyapatite Coating

**Figure 1:**
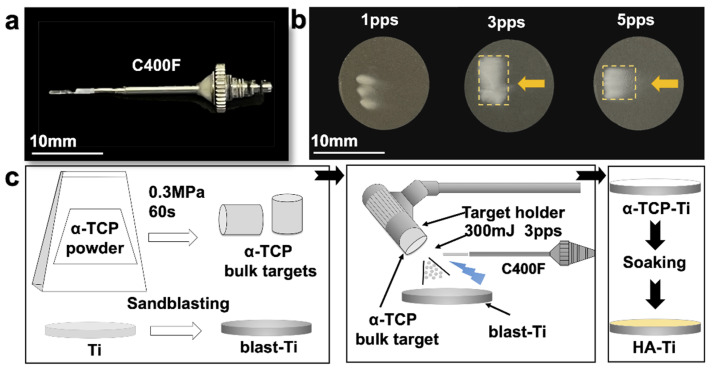
(**a**) The straightened C400F contact tip. (**b**) Gross appearances of the α-TCP coatings. (**c**) Fabrication procedure of the HA coating.

Ti discs (diameter, 15 mm; length, 1 mm) were used in the present study. The discs were polished for 1 min using SiC abrasive papers (800# and 1200#), followed by an MG400-CS micro grinding machine (Meiwafosis, Tokyo, Japan). The Ti discs were then sandblasted (blast-Ti) as a pretreatment method to increase their Ra. Following sandblasting, the discs were ultrasonically cleaned with acetone, ethanol, and deionized water for 10 min each, and then dried for 4 h in room-temperature air (20–25 °C). PLD was performed with an Er:YAG laser unit (Erwin AdvErl Unit; Morita Manufacturing, Kyoto, Japan) with a straightened C400F contact tip (Morita Manufacturing, retrofit) (Figure 1a). Details of the unit specifications are provided in a previous study [21]. Approximately 150 mg of α-TCP powder (Taihei Chemical Industrial, Osaka, Japan) was weighed and placed in a hydraulic press (0.3 MPa for 60 s) to form targets (diameter, 5 mm; thickness, 7 mm) for PLD. The α-TCP target was irradiated by the Er:YAG laser unit with a pulse energy of 300 mJ/pulse, under the same conditions applied in a previous study [17]. The gross appearance of α-TCP coatings deposited by Er:YAG-PLD with pulse frequencies of 1, 3, and 5 pps for 3 s is shown in Figure 1b. When the pulse frequency was 3 pps, the particle size of the powder was fairly small; when the pulse frequency was increased to 5 pps, the deposited range of separated particles was too large for controllable deposition. Thus, laser irradiation was conducted at a pulse energy of 300 mJ/pulse and a frequency of 3 pps in the present study. To achieve a megascopic α-TCP coating on the blast-Ti (α-TCP-Ti), we manually moved the Er:YAG-PLD handpiece horizontally. The coated Ti discs were then soaked in water and placed in a 90 °C incubator for 10 h [22] to obtain the HA-coated samples (HA-Ti). For in vitro experiments, all samples were sterilized in an autoclave at 160 °C for 3 h. The fabrication procedure of the HA coating is illustrated in Figure 1c.

### 2.2. Characterization

Scanning electron microscopy (SEM, S-4800; Hitachi, Tokyo, Japan) was used to examine the surface morphology and microstructure of the prepared materials. The SEM samples were coated with a very thin and conductive Os layer using an Os coating machine (HPC-20; Vacuum Device, Ibaraki, Japan). The mean average Ra and three-dimensional surface topography of the samples were assessed by scanning probe microscopy (SPM, SPM-9600; Shimadzu, Kyoto, Japan). X-ray photoelectron spectrometry (XPS, ESCA-5600; ULVAC-PHI, Kanagawa, Japan) was used to analyze the surface chemical states and elemental composition of the samples. Energy-dispersive X-ray spectroscopy (EDS, JED-2300, JEOL, Tokyo, Japan) was used to determine the elements present in the samples. X-ray diffractometry (XRD, Ultima IV, Rigaku, Tokyo, Japan) was used to determine the crystallinity of the coatings. The analysis was performed using Cu Kα radiation at 40 kV and 100 mA; the scan speed was 2°/min, the incidence angle was 1°, and the 2θ range was 3–80°. Fourier-transform infrared (FTIR) spectroscopy (IRAffinity-1S; Shimadzu) was used to establish the presence of two groups of phosphates (–PO_4_^3−^) found in the samples. The Z-axis pull test was used to measure the binding strength of the coatings. After adhering a stainless steel rod (diameter, 3 mm) to the coatings with epoxy glue, the sample was mounted to the jig of an all-purpose joint tensile tester (EZ Test, Shimadzu). Tensile loads were applied to the specimens at the rate of 0.5 mm/min until failure.

### 2.3. Cell Cultures

In this study, the femurs of 8-week-old male rats were harvested, and the rat BMMSCs (rBMMSCs) (Shimizu Laboratory Supplies Co., Kyoto, Japan) were cultured in a 75 cm^2^ flask with Eagle’s minimum essential medium (E-MEM; Nacalai Tesque, Inc., Kyoto, Japan) containing 10% fetal bovine serum (Nacalai Tesque, Inc.) and an antibiotic–antimycotic solution (Nacalai Tesque, Inc.) at 37 °C, according to the protocol described in a previous article [34]. In vitro investigations were conducted using cells from the third and fourth generations. After digestion with 0.5 g/L trypsin, the cells were resuspended and inoculated at a density of 5 × 10^4^ cells/well in 24-well plates containing Ti, TCP-Ti, and HA-Ti. The criteria of Osaka Dental University for animal testing were followed in this study (Approval No. 21-09002).

### 2.4. Attachment and Proliferation of Cells

Cell attachment and proliferation were determined using the CellTiter-Blue^®^ Cell Viability test kit (Promega, Madison, WI, USA) according to the manufacturer’s instructions. Cell attachment was determined at three time points of 3, 12, and 24 h, and cell proliferation was determined after 3, 7, and 14 d. The culture medium was removed, and the samples were washed twice with PBS and treated with 50 µL of CellTiter-Blue^®^ reagent diluted with 250 µL of PBS. After 1 h of culture at 37 °C in a 5% CO_2_ environment, 100 µL of the reagent was applied to each well of a 96-well plate. A microplate reader was used to determine the fluorescence of the solutions at 560 and 590 nm (SpectraMax M5; Molecular Devices, San Jose, CA, USA).

### 2.5. Morphology of Cells

Confocal laser scanning microscopy after fluorescent labeling and SEM after critical -point drying were used to examine cell morphology.

After a 24 h incubation period, the cells were stained with fluorescent dye. We removed the medium from the 24-well plates, rinsed the cells thrice with PBS, fixed them in 1 mL of 4% paraformaldehyde (PFA) solution, and then re-plated them. Next, 0.2% (*v/v*) Triton X-100 was added to the cells, which were subsequently incubated for 30 min at room temperature (20–25 °C) after the initial 20 min treatment. Thereafter, the cells were treated with Blocking One reagent for 30 min at room temperature and stained with Alexa Fluor^®^ 488 and 4′,6-diamidino-2-phenylindole at 37 °C in the dark for 1 h. The labeled cells were washed thrice with PBS. A final examination of actin filaments and cell nuclei was carried out using a confocal laser scanning microscope (LSM700; Carl Zeiss AG, Wetzlar, Germany).

The cell samples were processed for SEM to evaluate their pseudopodia and extracellular morphology. The culture medium was removed from the 24-well plates after 24 h of incubation. The cells were washed thrice with PBS and fixed for 2 h in 1 mL of a 4% PFA solution at 4 °C. The PFA solution was withdrawn, and the cells were rinsed thrice with PBS and successively dehydrated with a graded series of ethanol solutions (50%, 60%, 70%, 80%, 90%, and anhydrous) for 10 min each time. They were then dried in a critical-point dryer (HCP-1; Hitachi) and coated with Os using an ion-sputtering machine (HPC-20; Vacuum Device) for SEM examination after immersion in 3-methylbutyl acetate for 30 min (S-4800; Hitachi).

### 2.6. Statistical Analysis

The surface characterization and cell culture experiments were performed in triplicate. All quantitative data are presented as means ± SD. The results were analyzed by one-way analysis of variance and Bonferroni’s post hoc test using GraphPad Prism 8.0 software (GraphPad Prism, San Diego, CA, USA); *p* < 0.05 was regarded as statistically significant, while *p* < 0.01 was considered highly significant.

## 3. Results

### 3.1. Characterization of the Hydroxyapatite Coating

**Figure 2:**
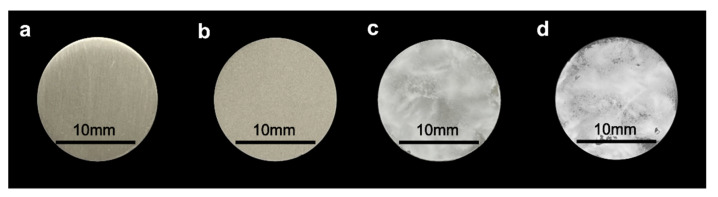
Gross appearance of (**a**) Ti, (**b**) blast-Ti, (**c**) α-tricalcium phosphate (α-TCP)-Ti, and (**d**) hydroxyapatite (HA)-Ti discs.

Figure 2 shows the gross appearance of Ti, blast-Ti, α-tricalcium phosphate (α-TCP)-Ti, and hydroxyapatite (HA)-Ti. The Ti surface (Figure 2a) appeared smooth and shiny; by contrast, the surface of blast-Ti (Figure 2b) was rough. The surfaces of α-TCP-Ti (Figure 2c) and HA-Ti (Figure 2d) were covered with a white film.

**Figure 3:**
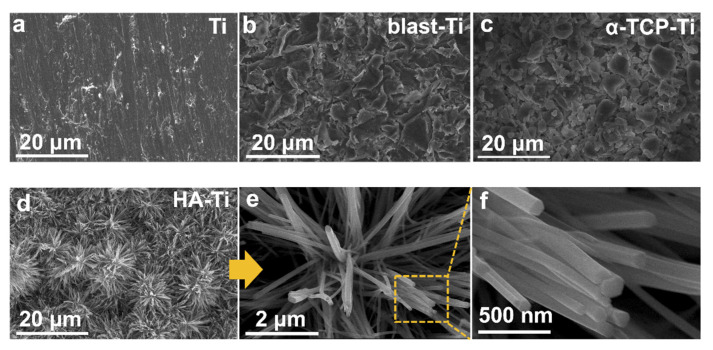
Scanning electron micrographs of (**a**) Ti, (**b**) blast-Ti, (**c**) α-TCP-Ti, and (**d**)–(**f**) HA-Ti discs. (**f**) Magnification of the area marked by the yellow rectangle in (**e**).

The SEM results clearly revealed that the HA coating could be obtained via the hydrothermal treatment of the α-TCP coating. The same phenomenon was observed in a previous study [30]. Figure 3a shows the micromorphology of commercial Ti. Figure 3b shows that the Ti surface became rough after sandblasting. Figure 3c shows the micromorphology of the α-TCP coating, which has a block structure. The HA coating, which is shown in Figure 3d, had a microstructure composed of nanorods with diameters of approximately 100 nm; this coating was obtained from the transformation of α-TCP. The cross-section of an HA crystal column is approximately hexagonal, as shown in Figure 3e,f.

**Figure 4:**
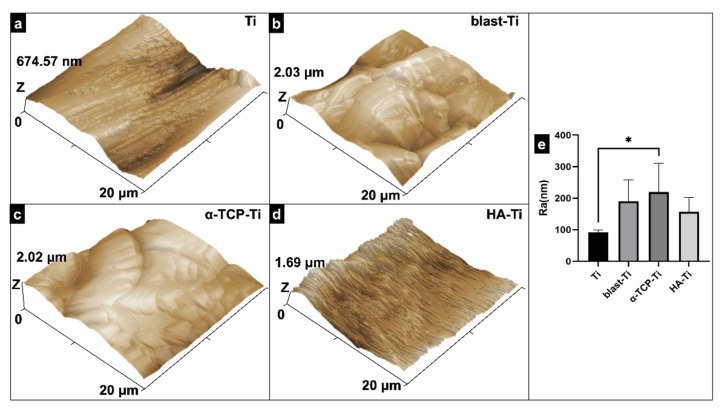
Scanning probe micrographs of (**a**) Ti, (**b**) blast-Ti, (**c**) α-TCP-Ti, and (**d**) HA-Ti discs. (**e**) Roughness (Ra) values of Ti, blast-Ti, α-TCP-Ti, and HA-Ti discs. The data shown are means ± SD (*n* = 3); * *p* < 0.05.

The topographical features of Ti, blast-Ti, α-TCP-Ti, and HA-Ti are shown in Figure 4a–d, respectively. Figure 4e reveals that the Ra values of blast-Ti and α-TCP-Ti are 190.14 and 219.41 nm, respectively, which are clearly higher than those of Ti (91.44 nm) and HA-Ti (156.88 nm). Moreover, the Ra of Ti was much lower than that of HA-Ti (* *p* < 0.05).

**Figure 5:**
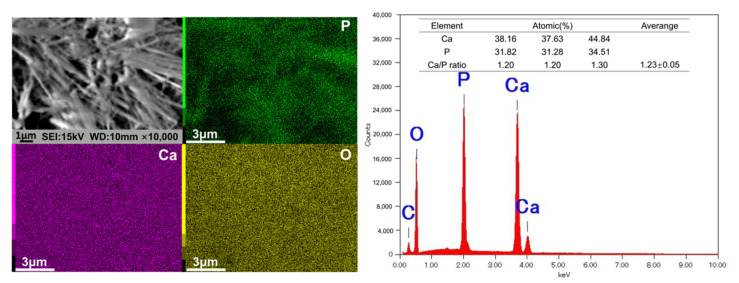
Energy-dispersive X-ray spectroscopy and elemental mapping (Ca, P, O) of the HA-Ti discs.

The exact stoichiometric phase formation of the samples was determined via EDS analysis, the results of which are depicted in Figure 5. The compositional analysis revealed the peaks of Ca, P, O, and C on the surface of HA-Ti. The atomic percentages of Ca and P and the ratio of Ca:P are shown in Figure 5. The average Ca:P ratio was 1.23 ± 0.05, which indicated that the HA coating on the HA-Ti sample was Ca-deficient. The results of elemental mapping showed that Ca, P, and O were uniformly distributed in the samples; a dense region along the rod-like structure of HA could also be observed.

**Figure 6:**
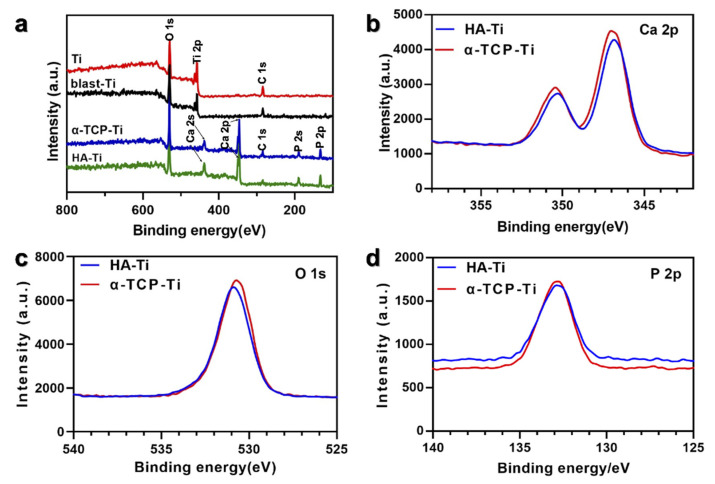
X-ray photoelectron spectra (XPS) of Ti, blast-Ti, α-TCP-Ti, and HA-Ti discs: (**a**) Wide-scan XPS of the material surfaces. (**b**) High-resolution spectra of Ca 2p. (**c**) High-resolution spectra of O 1s. (**d**) High-resolution spectra of P 2p.

The chemical compositions of Ti, blast-Ti, α-TCP-Ti, and HA-Ti are shown in Figure 6. The wide-scan XPS spectra (Figure 6a) of Ti and blast-Ti revealed the characteristic peaks of O 1s, Ti 2p, and C 1s; by comparison, the XPS spectra of α-TCP-Ti and HA-Ti revealed the characteristic peaks of O 1s, Ca 2p, Ca 2s, C 1s, P 2s, and P 2p. The O 1s peak was observed at 530.11 eV, the Ti 2p peak at 457.00 eV, the Ca 2p and Ca 2s peaks at 438.33 and 346.56 eV, respectively, the C 1s peak at 284.33 eV, and the P 2s and P 2p peaks at 189.44 and 135.00 eV, respectively. The high-resolution spectra of Ca 2p, O 1s, and P 2p are shown in Figure 6b–d, respectively.

**Figure 7:**
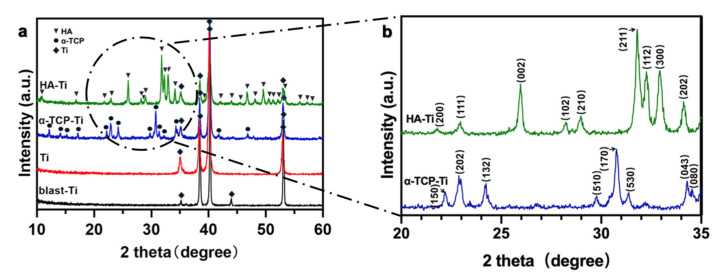
(**a**) X-ray diffraction patterns of Ti, blast-Ti, α-TCP-Ti, and HA-Ti discs. (**b**) Magnification of the spectra of α-TCP-Ti (blue) and HA-Ti (green) marked by the black circle in (**a**).

The XRD patterns of Ti, blast-Ti, α-TCP-Ti, and HA-Ti are shown in Figure 7a. The XRD measurements of the out-of-plane and in-plane geometries of the thin films were obtained to describe the crystal structures of the thin-film crystals, referred to HA (JCPDS NO.72-1243) and α-TCP (JCPDS NO.09-0348). In the in-plane XRD pattern shown in Figure 7b, the peaks observed at 20° and 35° are attributable to the (200), (111), (002), (102), (210), (211), (112), (300), and (202) reflections of HA and the (150), (202), (132), (510), (170), (530), (043), and (080) reflections of α-TCP, respectively. The XRD pattern of HA-Ti was consistent with that of standard HA. These results indicate that we had successfully prepared an HA coating on Ti.

**Figure 8:**
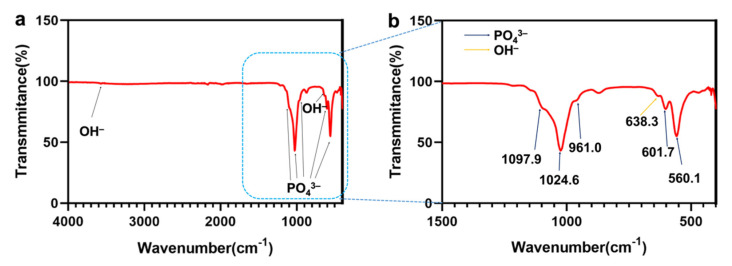
(**a**) FTIR spectrum of an HA-Ti disc. (**b**) Magnification of the spectrum marked by the blue rectangle in (**a**).

The FTIR spectra of the samples are shown in Figure 8. The bending vibration mode of –PO_4_^3−^ is responsible for the absorption bands at 1097.9, 1024.6, 961.0, 601.7, and 560.1 cm^−1^. The absorption bands of –OH^−^ could be observed at 3588.0 and 638.3 cm^−1^. These results confirm the successful formation of HA on the Ti discs.

**Figure 9:**
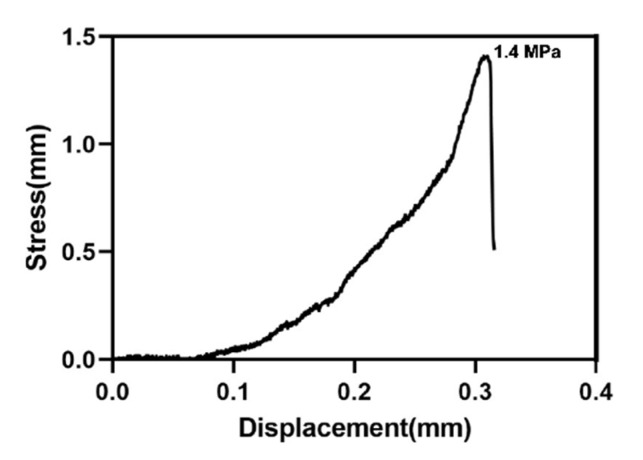
Stress–displacement curves for the deposited HA coating.

Figure 9 shows the tensile test results of the HA coating on the sandblasted Ti. The adhesive strength of the coating was approximately 1.4 MPa.

### 3.2. Attachment and Proliferation of Cells

**Figure 10:**
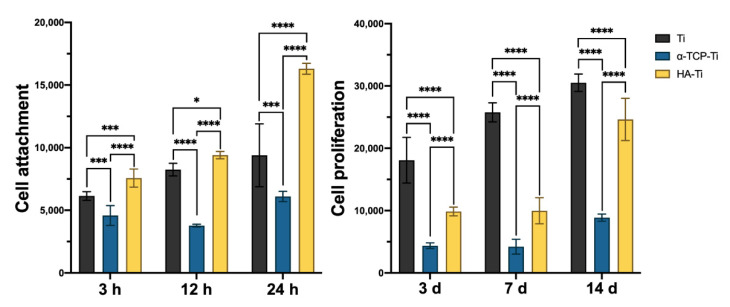
Cell attachment and proliferation of rat bone marrow mesenchymal stem cells on Ti, α-TCP-Ti, and HA-Ti discs (*n* = 3); * *p* < 0.05, *** *p* < 0.001, **** *p* < 0.0001.

Figure 10 shows the extent of cell growth on Ti, α-TCP-Ti, and HA-Ti. Compared with pure Ti incubated for 3 h, 12 h, 24 h, 3 d, 7 d, and 14 d, α-TCP-Ti exhibited an evident inhibitory effect on rBMMSC adhesion and growth. This finding indicates that α-TCP is cytotoxic to a certain extent. HA-Ti showed higher levels of cell attachment compared with the Ti discs at 3, 12, and 24 h; however, this sample showed lower levels of cell proliferation compared with Ti at 3, 7, and 14 d.

### 3.3. Morphology of Cells

**Figure 11:**
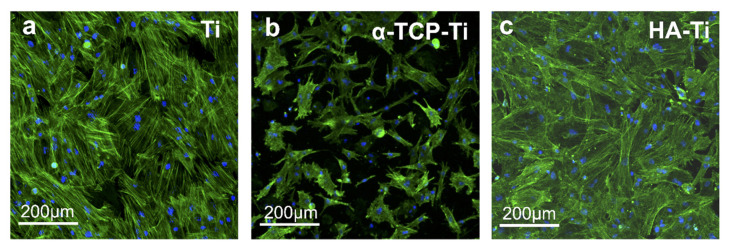
Morphological analysis of rat bone marrow mesenchymal stem cells attached to (**a**) Ti, (**b**) α-TCP-Ti, and (**c**) HA-Ti discs after 24 h of culture. Actin filaments (green) were labeled with Alexa Fluor^®^ 488, and nuclei (blue) were stained with 4′,6-diamidino-2-phenylindole.

The morphology of cells incubated for 24 h on Ti, α-TCP-Ti, and HA-Ti is shown in Figure 11. Compared with Ti and α-TCP-Ti, HA-Ti showed the largest number of adhered cells. The number of cells adhered on α-TCP-Ti was comparably less than that adhered on HA-Ti. Moreover, the pseudopodia of cells cultured on HA-Ti stretched extensively and in different directions.

**Figure 12:**
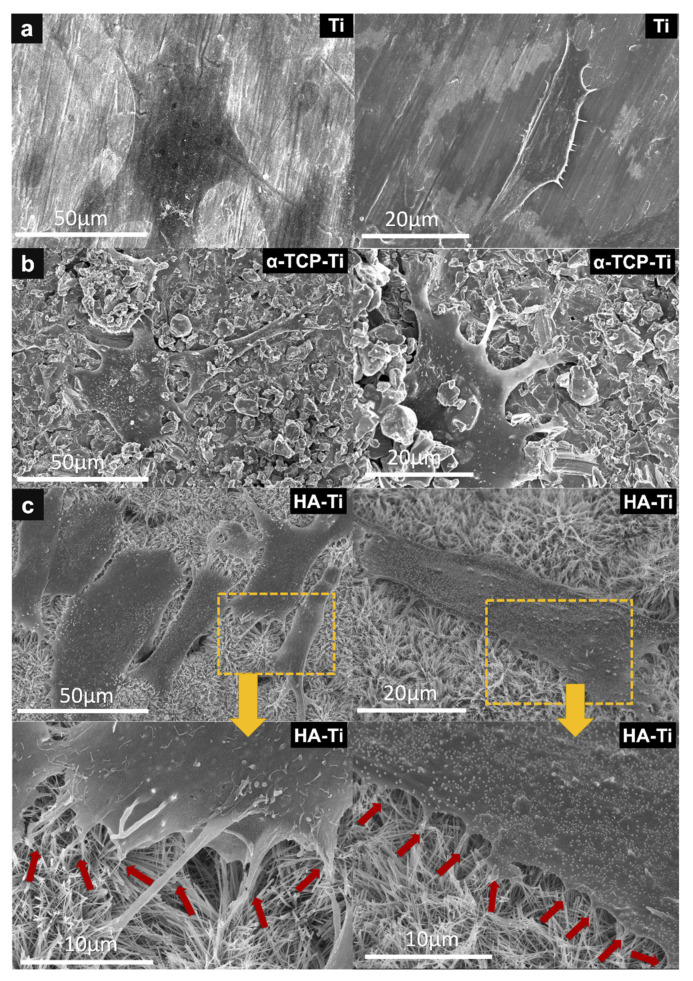
SEM of rat bone marrow mesenchymal stem cells attached to (**a**) Ti, (**b**) α-TCP-Ti, and (**c**) HA-Ti discs after 24 h of culture. Higher magnification images of the area marked by the orange rectangular area in (**c**) clearly revealed the morphology and number of cellular pseudopods on HA-Ti (marked by red arrows).

The morphology of the cells after 24 h of culture was examined via SEM. The cells attached and spread close to the surface of the specimens (Figure 12). Compared with those on Ti (Figure 12a) and blast-Ti (Figure 12b), the cells on HA-Ti (Figure 12c) had numerous cytoplasmic extensions and filopodia, and spread along the uneven surface of the sample. Higher magnification images of a specific area of the HA-Ti sample (marked by the orange rectangular area in Figure 12c) clearly revealed the morphology and number of cellular pseudopods (marked by red arrows).

## 4. Discussion

**Figure 13:**
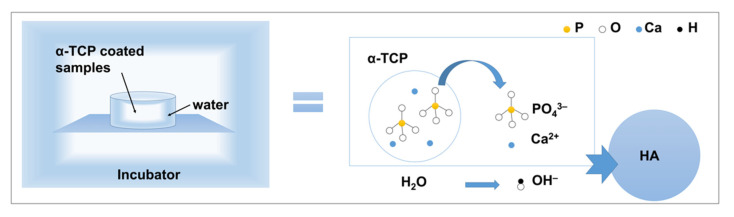
The schematics for hydrolysis of α-TCP to HA.

In the present study, Er:YAG-PLD was used to produce an α-TCP coating on sandblasted Ti discs. A nanorod-like HA coating was then successfully obtained via the hydrothermal treatment of the discs at 90 °C for 10 h. Previous research [24,32] has demonstrated that the hydrolysis of α-TCP is a dissolution and precipitation process; a schematic of the hydrolysis of α-TCP to HA is shown in Figure 13.

SEM confirmed the nanorod-like microstructure of HA, as confirmed by an earlier study, which demonstrated a typical flower-like morphology under low magnification [30]. The SPM results showed that the HA coating had a higher Ra compared with Ti, which is favorable for surface cell attachment [6,7]. Identical results were obtained by other authors [34]. Although the Ra of blast-Ti and α-TCP-Ti was higher than that of Ti and HA-Ti, bone healing around an implant also depends on other factors, such as surface topography and chemical composition, which influence cell reactions. The elemental composition of the coatings was determined by EDS and XPS, and Ca, P, and O were detected. We found that the Ca:P ratio was low; the reason(s) for this this could be investigated in a future study. The results of XRD and FTIR confirmed that the HA coating had been successfully prepared on the Ti discs. The microstructure, elemental composition, object–image composition, and functional group structure results collectively confirmed the successful deposition of an HA film on the surface of the Ti discs. Because the bonding strength of the implant surface coating can affect the osseointegration between the implant and bone tissue, we evaluated the mechanical properties of the coating via a tensile test. According to this study [35], the bonding strength between HA-coated titanium and bone tissue is ~3.5 MPa. Further optimization of the experimental conditions is necessary in order to enhance the coating stability of HA.

rBMMSCs were cultured on the coated discs to evaluate their cell behavior. Previous studies showed that nano/microstructured HA has a similar composition and structure to natural bone tissue [36,37,38,39], and can obviously promote the adhesion, proliferation, differentiation, and mineralization of osteoblasts [40]. Nano/microstructured surfaces can enhance the osteogenic differentiation of osteoblasts, even in the absence of osteogenic supplements [41,42]. Based on these studies, we speculated that cell attachment on the HA-coated specimens would be more extensive than that in the other groups after 24 h of culture, owing to the nanorod-like morphology and roughness of HA; however, we observed that the cell viability of the HA-Ti samples on days 3, 7, and 14 was lower than that of the control group. This finding may be related to the dissolution of the HA film; previous studies have confirmed that HA can easily be dissolved [43]. The morphology of the cultured cells was observed by fluorescence staining and SEM. rBMMSCs cultured on the HA-Ti samples clearly exhibited good adhesion and spreading, showing numerous cytoplasmic extensions and filopodia, and interconnections along their uneven surface. These results suggest that HA-Ti is beneficial for cell growth. A similar phenomenon was observed in an earlier study [44].

Er:YAG-PLD could be used to produce HA coatings with a nanorod-like morphology on Ti. This surface modification technique may be useful to obtain microstructured coating materials for implant surfaces. HA coatings are used as bioactive materials that can not only directly promote osteogenesis, but also induce bone differentiation [45,46]. Further cell differentiation experiments with HA coatings should be undertaken. The acid resistance and stability of the films also requires further research and evaluation. In future work, we will prepare HA films on sandblasted implant surfaces and evaluate their osteogenic potential via in vivo experiments.

## 5. Conclusions

In summary, surface modification is required in order to endow implant surfaces with bioactivity and improve their osteogenesis.

In this study, Er:YAG PLD was used as a pretreatment method, and α-TCP was used as a precursor. An HA coating with a nanorod-like structure was obtained via the hydrothermal treatment of α-TCP-coated Ti discs at 90 °C for 10 h. The prepared HA coating showed a certain degree of roughness, and the XRD and FTIR results indicated that the degree of α-TCP hydrolysis was relatively complete under the reaction conditions employed in this study.

The HA coating promoted better cell adhesion and expansion on the Ti surface, owing to its nanostructure and biological activity, which are beneficial for endowing implant surfaces with good biocompatibility.

Because the HA coating is affected by many factors, methods to develop stable HA coatings should be explored in future work. The osteogenic response in implants is a complex process. Thus, osteogenic differentiation experiments and in vivo experiments are needed in order to verify the osteogenic effect of HA coatings on Ti implants.

## Data Availability

Not applicable.

## References

[1] Jemat A., Ghazali M.J., Razali M., Otsuka Y. (2015). Surface Modifications and Their Effects on Titanium Dental Implants. BioMed Res. Int..

[2] Ottria L., Lauritano D., Andreasi Bassi M., Palmieri A., Candotto V., Tagliabue A., Tettamanti L. (2018). Mechanical, Chemical and Biological Aspects of Titanium and Titanium Alloys in Implant Dentistry. J. Biol. Regul. Homeost. Agents.

[3] Xuereb M., Camilleri J., Attard N.J. (2015). Systematic Review of Current Dental Implant Coating Materials and Novel Coating Techniques. Int. J. Prosthodont..

[4] Puleo D.A., Nanci A. (1999). Understanding and Controlling the Bone-Implant Interface. Biomaterials.

[5] Bosshardt D.D., Chappuis V., Buser D. (2017). Osseointegration of Titanium, Titanium Alloy and Zirconia Dental Implants: Current Knowledge and Open Questions. Periodontology 2000.

[6] Kern M., Thompson V.P. (1994). Effects of Sandblasting and Silica-Coating Procedures on Pure Titanium. J. Dent..

[7] Annunziata M., Guida L., Perillo L., Aversa R., Passaro I., Oliva A. (2008). Biological Response of Human Bone Marrow Stromal Cells to Sandblasted Titanium Nitride-Coated Implant Surfaces. J. Mater. Sci. Mater. Med..

[8] Ryu J., Kang T., Shin H., Kim K. (2020). Osteogenic Properties of Novel Hydroxyapatite Scaffold. Int. J. Mol. Sci..

[9] Guillen-Romero L.D., Oropeza-Guzmán M.T., López-Maldonado E.A., Iglesias A.L., Paz-González J.A., Ng T., Serena-Gómez E., Villarreal-Gómez L.J. (2019). Synthetic Hydroxyapatite and Its Use in Bioactive Coatings. J. Appl. Biomater. Funct. Mater..

[10] Uskoković V., Uskoković D.P. (2011). Nanosized Hydroxyapatite and Other Calcium Phosphates: Chemistry of Formation and Application as Drug and Gene Delivery Agents. J. Biomed. Mater. Res. B Appl. Biomater..

[11] Sun F., Zhou H., Lee J. (2011). Various Preparation Methods of Highly Porous Hydroxyapatite/Polymer Nanoscale Biocomposites for Bone Regeneration. Acta Biomater..

[12] Kumar A., Biswas K., Basu B. (2015). Hydroxyapatite–Titanium Bulk Composites for Bone Tissue Engineering Applications. J. Biomed. Mater. Res. A.

[13] Kreisler M., Kohnen W., Marinello C., Götz H., Duschner H., Jansen B., d’Hoedt B. (2002). Bactericidal Effect of the Er:YAG Laser on Dental Implant Surfaces: An In Vitro Study. J. Periodontol..

[14] AlMoharib H.S., Steffensen B., Zoukhri D., Finkelman M., Gyurko R. (2021). Efficacy of an Er:YAG Laser in the Decontamination of Dental Implant Surfaces: An In Vitro Study. J. Periodontol..

[15] Hympanova L., Mackova K., El-Domyati M., Vodegel E., Roovers J.P., Bosteels J., Krofta L., Deprest J. (2020). Effects of Non-Ablative Er:YAG Laser on the Skin and the Vaginal Wall: Systematic Review of the Clinical and Experimental Literature. Int. Urogynecol. J..

[16] Kriechbaumer L.K., Happak W., Distelmaier K., Thalhammer G., Kaiser G., Kugler S., Tan Y., Leonhard M., Zatorska B., Presterl E. (2020). Disinfection of Contaminated Metal Implants with an Er:YAG Laser. J. Orthop. Res..

[17] Chen L., Hontsu S., Komasa S., Yamamoto E., Hashimoto Y., Matsumoto Y. (2021). Hydroxyapatite Film Coating by Er:YAG Pulsed Laser Deposition Method for the Repair of Enamel Defects. Materials.

[18] Hontsu S., Kato N., Yamamoto E., Yoshikawa K., Hashimoto Y. (2014). Fabrication of a HA Films Using the Er: YAG Laser. J. Bio-Integr. Bio Integr. Soc..

[19] Hontsu S. (2000). Preparation of Biomaterial Thin Films by Pulsed-Laser Deposition Technique. Rev. Laser Eng..

[20] Xiao Y., Li Y.L., Gao H.Y., Wang G.L., Xia P. (2016). Alpha-Tricalcium Phosphate as a Bone Graft: Research and Development in Orthopaedics. Chin. J. Tissue Eng. Res..

[21] Ma Y., Li S.-W., Feng Z.-D. (2009). Restoration of Tooth Enamel Caries by Hydrolysis of α-TCP. J. Inorg. Mater..

[22] Park Y.M., Yang T.Y., Yoon S.Y., Stevens R., Park H.C. (2005). Hydrolysis of α-TCP and Development of Hydroxyapatite Whiskers. Key Eng. Mater..

[23] Fernández E., Gil F.J., Ginebra M.P., Driessens F.C., Planell J.A., Best S.M. (1999). Calcium Phosphate Bone Cement for Clinical Applications. Part I: Solution Chemistry. J. Mater. Sci. Mater. Med..

[24] Brown P.W. (1999). Hydration Behavior of Calcium Phosphates is Analogous to Hydration Behavior of Calcium Silicates. Cem. Concr. Res..

[25] Ginebra M.-P., Driessens F.C.M., Planell J.A. (2004). Effect of the Particle Size on the Micro and Nanostructural Features of a Calcium Phosphate Cement: A Kinetic Analysis. Biomaterials.

[26] Ginebra M.-P., Fernández E., Driessens F.C.M., Planell J.A. (1999). Modeling of the Hydrolysis of α-Tricalcium Phosphate. J. Am. Ceram. Soc..

[27] Takeuchi A., Munar M.L., Wakae H., Maruta M., Matsuya S., Tsuru K., Ishikawa K. (2009). Effect of Temperature on Crystallinity of Carbonate Apatite Foam Prepared from α-Tricalcium Phosphate by Hydrothermal Treatment. Biomed. Mater. Eng..

[28] Yoshimura M., Suda H., Okamoto K., Ioku K. (1994). Hydrothermal Synthesis of Biocompatible Whiskers. J. Mater. Sci..

[29] Durucan C., Brown P.W. (2002). Kinetic Model for α–Tricalcium Phosphate Hydrolysis. J. Am. Ceram. Soc..

[30] Ma M.-G. (2012). Hierarchically Nanostructured Hydroxyapatite: Hydrothermal Synthesis, Morphology Control, Growth Mechanism, and Biological Activity. Int. J. Nanomed..

[31] Charbord P. (2010). Bone Marrow Mesenchymal Stem Cells: Historical Overview and Concepts. Hum. Gene Ther..

[32] Chen L., Komasa S., Hashimoto Y., Hontsu S., Okazaki J. (2018). In Vitro and In Vivo Osteogenic Activity of Titanium Implants Coated by Pulsed Laser Deposition with a Thin Film of Fluoridated Hydroxyapatite. Int. J. Mol. Sci..

[33] Zhang H., Komasa S., Mashimo C., Sekino T., Okazaki J. (2017). Effect of Ultraviolet Treatment on Bacterial Attachment and Osteogenic Activity to Alkali-Treated Titanium with Nanonetwork Structures. Int. J. Nanomedicine..

[34] Xu Y., Li H., Wu J., Yang Q., Jiang D., Qiao B. (2018). Polydopamine-induced Hydroxyapatite Coating Facilitates Hydroxyapatite/Polyamide 66 Implant Osteogenesis: An In Vitro and In Vivo Evaluation. Int. J. Nanomedicine..

[35] Kim J., Kwun S.I., Yoon J.G. (1998). Electrical Properties of Nb-Doped SrTiO_3_ Thin Films Grown by Pulsed Laser Deposition. J. Korean Phys. Soc..

[36] Gupta D., Venugopal J., Mitra S., Dev V.R.G., Ramakrishna S. (2009). Nanostructured Biocomposite Substrates by Electrospinning and Electrospraying for the Mineralization of Osteoblasts. Biomaterials.

[37] Jang J.-H., Castano O., Kim H.-W. (2009). Electrospun Materials as Potential Platforms for Bone Tissue Engineering. Adv. Drug Deliv. Rev..

[38] Han Y., Zhou J., Lu S., Zhang L. (2013). Enhanced Osteoblast Functions of Narrow Interligand Spaced Sr-HA Nanofibers/rods Grown on Microporous Titania Coatings. RSC Adv..

[39] Han Y., Zhou J., Zhang L., Xu K. (2011). A Multi-Scaled Hybrid Orthopedic Implant: Bone ECM-Shaped Sr-HA Nanofibers on the Microporous Walls of a Macroporous Titanium Scaffold. Nanotechnology.

[40] Ma X.P. (2008). Biomimetic Materials for Tissue Engineering. Adv. Drug Deliv. Rev..

[41] Dalby M.J., Gadegaard N., Tare R., Andar A., Riehle M.O., Herzyk P., Wilkinson C.D., Oreffo R.O. (2007). The Control of Human Mesenchymal Cell Differentiation Using Nanoscale Symmetry and Disorder. Nat. Mater..

[42] Kuo S.W., Lin H.-I., Ho J.H.-C., Shih Y.R., Chen H.F., Yen T.J., Lee O.K. (2012). Regulation of the Fate of Human Mesenchymal Stem Cells by Mechanical and Stereo-Topographical Cues Provided by Silicon Nanowires. Biomaterials.

[43] Hashimoto Y., Ueda M., Kohiga Y., Imura K., Hontsu S. (2018). Application of fluoridated hydroxyapatite thin film coatings using KrF pulsed laser deposition. Dent. Mater. J..

[44] Lukaszewska-Kuska M., Wirstlein P., Majchrowski R., Dorocka-Bobkowska B. (2018). Osteoblastic cell behaviour on modified titanium surfaces. Micron.

[45] Łukaszewska-Kuska M., Krawczyk P., Martyla A., Hędzelek W., Dorocka-Bobkowska B. (2018). Hydroxyapatite Coating on Titanium Endosseous Implants for Improved Osseointegration: Physical and Chemical Considerations. Adv. Clin. Exp. Med..

[46] Shi J., Dong L.L., He F., Zhao S., Yang G.-L. (2013). Osteoblast responses to thin nanohydroxyapatite coated on roughened titanium surfaces deposited by an electrochemical process. Oral Sur. Oral Med. Oral Pathol. Oral Radiol..

